# Protective effects of insulin on dry eye syndrome via TLR4/NF-κB pathway: based on network pharmacology and *in vitro* experiments validation

**DOI:** 10.3389/fphar.2024.1449985

**Published:** 2024-08-28

**Authors:** Xiuxiu Yuan, Yu Zhang, Siyi Wang, Zhiyu Du

**Affiliations:** ^1^ Ophthalmology Department, The Second Affiliated Hospital of Chongqing Medical University, Chongqing, China; ^2^ Chongqing Key Laboratory of ophthalmology, Chongqing, China; ^3^ Second Affiliated Hospital of Chongqing Medical University, Chongqing, China

**Keywords:** dry eye syndrome, insulin, network pharmacology, cell experiments, toll-like receptor signaling pathway

## Abstract

Dry eye syndrome (DES) is a multifactorial ocular surface disease and represents one of the most prevalent ophthalmic disorders. Insulin is an important metabolism-regulating hormone and a potential antioxidant with critical biological roles as anti-inflammatory and anti-apoptotic. However, its mechanism of action remains unknown. In this study, we used network pharmacology techniques and conducted cell experiments to investigate the protective effect of insulin on human corneal epithelial cells (HCECs). Eighty-seven common targets of insulin and DES were identified from the database. KEGG pathway enrichment analysis suggested that insulin may be crucial in regulating the toll-like receptor (TLR) signaling pathway by targeting key targets such as IL-6 and TNF. In cell experiments, insulin promoted HCECs proliferation, improved their ability to migrate, and inhibited apoptosis. Western blot and enzyme-linked immunosorbent assay (ELISA) also confirmed the upregulation of the expression of inflammatory factors such as IL-1β, IL-6, and proteins related to the TLR4/NF-κB signaling pathway. However, the expression of these proteins was inhibited by insulin administration. Our results preliminarily verified insulin may exert a protective role on HCECs under hyperosmotic condition, which offered a novel perspective for the clinical management of this condition.

## 1 Introduction

Dry Eye Syndrome (DES) is a prevalent ocular surface disease that affects millions of individuals annually and has a serious impact on the lives, studies, and work of many people (Chen et al., 2021). An increasing body of research indicates that inflammation plays a crucial role in the pathogenesis of DES, and it has been demonstrated to be triggered by hyperosmolarity of the tear film (Jin et al., 2020). The immunomodulatory mechanism of the ocular surface of DES patients is disordered, and numerous studies have shown the pivotal role of toll-like receptors (TLRs) and their signaling system in the inflammatory response of DES (Kiripolsky et al., 2020). Moreover, upregulation of this signaling pathway induces and exacerbates inflammation (Sahoo, 2020).

Artificial tears are the preferred clinical treatment for DES (Moshirfar et al., 2014; Sheppard et al., 2023), but it should be noted that if artificial tears contain preservative ingredients, in the case of frequent use, the patient’s ocular surface reaction is too large, it must be discontinued in a timely manner, and the use of artificial tears in patients with moderate-to-severe dry eye is not obvious.

Insulin is a biologically active polypeptide with a proven role as a protein hormone that promotes the synthesis of glycogen, lipids and proteins. In addition, it stimulates the migration of human epidermal cells, and topical insulin application has previously been shown to benefit the healing of ulcers and burns (Yang et al., 2020). Insulin also inhibits the production of reactive oxygen species by neuronal mitochondria in normal rats and attenuates oxidative stress injury in septic rats (Chen et al., 2014). It has also been noted that insulin normalizes mitochondrial morphology, protein synthesis and function in hepatocytes of patients with hyperglycemia (Vanhorebeek et al., 2005).

In recent years, a large number of experiments have confirmed that insulin also has anti-inflammatory and anti-apoptotic important biological significance (Conart et al., 2020; Ferreira et al., 2020; Liao et al., 2022; Pan et al., 2023). It can effectively inhibit the production of pro-inflammatory cytokines, inhibit the activation of NLRP3 inflammasome, and ultimately inhibit apoptosis (Chang et al., 2021). A study has demonstrated topical use of insulin has an effect on DES in diabetic rats (Cruz-Cazarim et al., 2019). However, the mechanism of action of insulin on DES is not clear.

Network pharmacology is an interdisciplinary field investigating drug action mechanisms and guiding clinical development. It encompasses various disciplines such as pharmacology, systems biology, bioinformatics, network science, and related fields (An et al., 2022). It aims to investigate the intricate balance within biological networks (Shang et al., 2023). It can be used to analyze the effects of drugs on the human body at a systematic level, which helps us to find the targets of drug action and improve the efficacy of drugs (Kong et al., 2021). In this study, we created an *in vitro* model of cellular hyperosmolarity to mimic the ocular environment of patients with DES, used network pharmacology to analyze the possible targets and molecular mechanisms of the action of insulin on DES, and verified the protective effect of insulin into this cellular model.

## 2 Materials and methods

### 2.1 Analysis based on network pharmacology

#### 2.1.1 Gene collection of DES

In GeneCards (https://www.genecards.org/, accessed on 15 July 2023) and CTD (https://ctdbase.org/, accessed on 15 July 2023) databases, we searched for genes related to DES by typing in “dry eye syndrome,” sorted and screened the acquired target genes, and deleted the duplicate sequences.

#### 2.1.2 Prediction of potential insulin targets

The SMILE structure of insulin was retrieved from the PubChem database (https://pubchem.ncbi.nlm.nih.gov/, accessed on 18 July 2023) and entered into the SEA database (https://sea.bkslab.org/, accessed on 18 July 2023) after searching the GeneCards database (https://www.genecards.org/, accessed on 18 July 2023) with insulin as the keyword, we organized and screened the acquired targets, deleting any duplicate sequences.

#### 2.1.3 Insulin-DES common targets screening and construction of the insulin-target-DES network diagram

We employed a Venn diagram (https://bioinfogp.cnb.csic.es/tools/venny/, accessed on 20 July 2023) to represent the overlapping targets of insulin and DES visually. Subsequently, we constructed a network diagram illustrating the interactions between insulin, targets, and DES using Cytoscape software (version 3.9.1, https://cytoscape.org/, accessed on 20 July 2023).

#### 2.1.4 PPI network construction

We utilized the STRING (version 12.0, https://www.string-db.org/, accessed on 23 July 2023) to construct the PPI network by submitting common targets in the multi-protein module, downloading the TSV format file, and importing it into Cytoscape software (version 3.9.1, https://cytoscape.org/, accessed on 23 July 2023) for common target PPI network visualization.

#### 2.1.5 GO functional annotation and KEGG pathway enrichment analysis

We performed GO functional annotation and KEGG pathway enrichment analysis of potential targets of insulin for DES using the Microbiotics platform (https://www.bioinformatics.com.cn/, accessed on 23 July 2023). GO functional annotation annotated gene function through three modules, BP, MF, and CC, we created bar graphs to depict the results of GO functional annotation, and bubble plots to show the results of KEGG pathway enrichment analysis. Additionally, we also created chord diagrams to visualize enrichment pathways and targets.

### 2.2 Experimental verification

#### 2.2.1 Cell lines and treatments

The immortalized human corneal epithelial cells (HCECs) were purchased from Bei Na Cell Bank (Beijing, China) and authenticated by STR. HCECs were cultured in DMEM (Gibco, California, United States) with 10% FBS (Gibco, California, United States), incubated at 37°C in a 5% CO_2_ incubator, and passaged routinely with 0.25% trypsin (Gibco, California, United States) at 80%–90% confluence. HCECs were divided into four groups (control, hyperosmolarity, hyperosmolarity-insulin co-culture, and insulin-alone groups). The control group was treated with a complete medium, while the hyperosmolarity group was exposed to a medium with a hyperosmolarity of 500 mOsM. The hyperosmolarity and insulin co-culture group was first treated with insulin at a concentration of 200 μg/mL and then exposed to a medium with a hyperosmolarity of 500 mOsM. Finally, the insulin-alone group was treated only with insulin at a concentration of 200 μg/mL. The detection indexes were as follows.

#### 2.2.2 Cell viability assay

The viability of HCECs was assessed using the cell counting kit-8 (CCK-8) assay kit (NCM, Suzhou, China, cat. no. C6005). HCECs were seeded in 96-well plates at a density of 1 × 10^4^ cells per well and incubated overnight. After that, HCECs were cocultured with different concentrations of NaCl (Sigma-Aldrich, St. Louis, Missouri, United States), including 0, 50, 70, 90, and 120 mM (the corresponding osmotic pressures being 312, 400, 450, 500, and 550 mOsM), or insulin (Sigma-Aldrich, St. Louis, Missouri, United States) at 10–500 μg/mL. Subsequently, the cells were incubated with a 10% CCK-8 kit for 2 h, and the absorption (OD value) was read at 450 nm.

#### 2.2.3 Cell cycle assay

The distribution of the cells in the different phases was measured using flow cytometry. Each group of cells was washed twice with PBS, and centrifuged at 8,000 rpm for 5 min, The cells were then fixed in pre-cooled 70% ethanol at −20°C overnight. The next day, the fixed cells were washed with PBS and incubated with 400 μL of PI/RNase (Beyotime, Shanghai, China) staining buffer for 15 min at room temperature. The analysis was conducted using flow cytometry (ThermoFisher, Massachusetts, United States). Cell cycle distribution rate was calculated using ImageJ software (version v1.8.0; National Institutes of Health).

#### 2.2.4 Scratch assay

HCECs were seeded in 6-well plates at a density of 1 × 10^6^cells per well and cultured for 24 h, the cells were scratched with a 200 µL pipette tip. Then, cells were washed three times with PBS to remove detached cells. Subsequently, images were acquired at 0, 24, and 48 h using an inverted microscope (Leica, Wetzler in Hesse, Germany). Wound healing rate was calculated using ImageJ software (version v1.8.0; National Institutes of Health).

#### 2.2.5 Transwell migration assay

The cell migration assay was performed using a 24-well transwell chamber. HCECs were seeded at a density of 3 × 10^4^ cells per well into the upper chamber (pore size, 8 μm), and the lower chamber was treated with different drugs, incubated at 37°C for 12–24 h, Then, cells on the upper chamber were fixed with 4% paraformaldehyde and stained with 0.1% crystal violet solution. After that, migration cells were observed using an inverted microscopy (Leica, Wetzler in Hesse, Germany). Migration cells number was calculated using ImageJ software (version v1.8.0; National Institutes of Health).

#### 2.2.6 Detection of reactive oxygen species (ROS) in cells

We used the dichloro-dihydro fluorescein diacetate (DCFH-DA) assay (Beyotime, Shanghai, China, cat. no. S0033S) to measure ROS levels in HCECs. HCECs were seeded in 6-well plates at a density of 5 × 10^5^ per well and cultured overnight. After that, cells were incubated for 20 min with 10 μM DCFH-DA dissolved in DMEM without FBS at 37°C in the dark. Fluorescence intensity was measured using an inverted fluorescence microscope (Leica, Wetzler in Hesse, Germany).

#### 2.2.7 Mitochondrial membrane potential assay

We measured the mitochondrial membrane potential (MMP) using a mitochondrial membrane potential assay kit with JC-1 (Beyotime, Shanghai, China, cat. no. C2003S). HCECs were seeded into the 6-well plates at a density of 5 × 10^5^ cells per well and cultured overnight. Subsequently, the cells were stained with JC-1 dye at 37°C for 20 min and washed twice with JC-1 staining buffer. Finally, the stained cells were observed using an inverted fluorescence microscope (Leica, Wetzler in Hesse, Germany).

#### 2.2.8 Calcein/PI staining

HCECs were seeded in 24-well plates (5 × 10^4^ cells/well) and incubated overnight. Each group of cells was stained with a Calcein/PI staining kit (Beyotime, Shanghai, China, cat. no. C2015S). Live cells were labeled green, and dead cells were marked red. Images were taken using an inverted fluorescence microscope (Leica, Wetzler in Hesse, Germany).

#### 2.2.9 Enzyme-linked immunosorbent assay (ELISA)

Inflammatory factors IL-1β, IL-6, and IL-8 levels were measured with ELISA kits (eBioscience, San Diego, CA, United States, cat. no. RK05046, RK00004, RK00011). The cell culture medium of HCECs treated with different drugs was collected, centrifuged, and then tested for the levels of inflammatory factors according to the manufacturer’s instructions.

#### 2.2.10 Immunofluorescence staining

HCECs were added in 24-well plates and cultured to cell adherence, fixed with 4% paraformaldehyde for 15 min, then permeabilized with 0.5% Triton X-100 for 20 min and blocked with 10% goat serum for 30 min, followed by incubation with monoclonal antibodies against HMGB1 (1:200; Abcam, Massachusetts, United States; cat. no. ab79823) and TNF-α (1:200; Abcam, Massachusetts, United States; cat. no. ab183218) at 4°C overnight. Afterward, cells were treated with the goat anti-rabbit IgG (H+L)Alexa Fluor^®^ 488) antibody (1:500; Abcam, Massachusetts, United States; cat. no. ab150077) at 37°C for 1 h, and the nuclei were counterstained with DAPI for 5 min. Finally, images were observed with an inverted fluorescence microscope (Leica, Wetzler in Hesse, Germany).

#### 2.2.11 Western blot

Total proteins of HCECs were extracted using rapid lysis buffer (Solarbio, Beijing, China). Subsequently, lysates were collected and centrifuged at 4°C for 15 min, protein concentrations of supernatants were measured by BCA assay (Beyotime, Shanghai, China, cat. no. P0011), proteins were mixed with SDS loading buffer in a 4:1 ratio and then boiled for 10 min. Equal amounts of protein (20 μg) were loaded onto a 4%–20% sodium dodecyl sulfate-polyacrylamide gel electrophoresis (SDS-PAGE) and transferred to polyvinylidene difluoride (PVDF) membranes. Following blocking the membranes with 5% skimmed milk for 2 h at room temperature, and the membranes were incubated with primary antibodies at 4°C overnight. The following primary antibodies were used: Anti-Bax (1:1000; Abcam, Massachusetts, United States; cat. no. ab32503), anti-Bcl-2 (1:1000; Abcam, Massachusetts, United States cat. no. ab182858), anti-GAPDH (1:1000; Abcam, Massachusetts, United States; cat. no. ab181602), anti-TLR4 (1:1000; Abcam, Massachusetts, United States; cat. no. ab218987), anti-MyD88 (1:1000; Abcam, Massachusetts, United States; cat. no. ab133739), anti-Tirap (1:1000; Abcam, Massachusetts, United States; cat. no. ab17218), anti-IKK-α (1:1000; Abcam, Massachusetts, United States; cat. no. ab32041), anti-p-IKK-α (1:1000; Abcam, Massachusetts, United States; cat. no. ab38515), anti-NF-κB p65 (1:1000; Abcam, Massachusetts, United States; cat. no. ab207297), anti-p-NF-κB p65 (1:1000; Abcam, Massachusetts, United States; cat. no. ab239882), anti-IκB-α (1:1000; Abcam, Massachusetts, United States; cat. no. ab32518), anti-p-IκB-α (1:1000; Abcam, Massachusetts, United States; cat. no. ab92700). Then, they were incubated with HRP-conjugated Goat anti-rabbit IgG antibody (1:5000; Abcam, Massachusetts, United States; cat. no. ab6721) at room temperature for 2 h. Finally, each membrane was developed using a chemiluminescence (ECL) detection kit (Beyotime, Shanghai, China, cat. no. P0018S) and visualized using a chemiluminescence detection system (VILBER, Paris, France). The gray value of the target bands was calculated using Image. J software (version v1.8.0; National Institutes of Health).

#### 2.2.12 Statistical analysis

Data analysis was performed using GraphPad Prism 8.0 (California, United States). The data were presented as means ± standard deviation (SD) based on a minimum of three replicates. Differences between groups were analyzed by one-way analysis of variance (ANOVA, Dunnett’s t-test). Significance was set up at *p* < 0.05.

## 3 Results

### 3.1 Analysis based on network pharmacology

#### 3.1.1 Prediction of DES targets

A total of 14,979 targets for DES were obtained from the CTD database, and 4,378 targets were identified from the GeneCards database, each with duplicate values deleted, and then the targets were standardized using the Uniprot database, a total of 668 targets with a selection score of 20 or more were selected.

#### 3.1.2 Prediction of insulin targets

After obtaining the SMILE structure of insulin from the PubChem database, we imported it into the SEA database for prediction and obtained 80 potential targets of insulin. Meanwhile, we collected 11,861 targets in the GeneCards database and standardized the targets through the UniProt database. 245 unique targets with a score of 20 or more were selected after deleting duplicate values.

#### 3.1.3 Construction of venny diagram and insulin-target-DES network diagram

**FIGURE 1:**
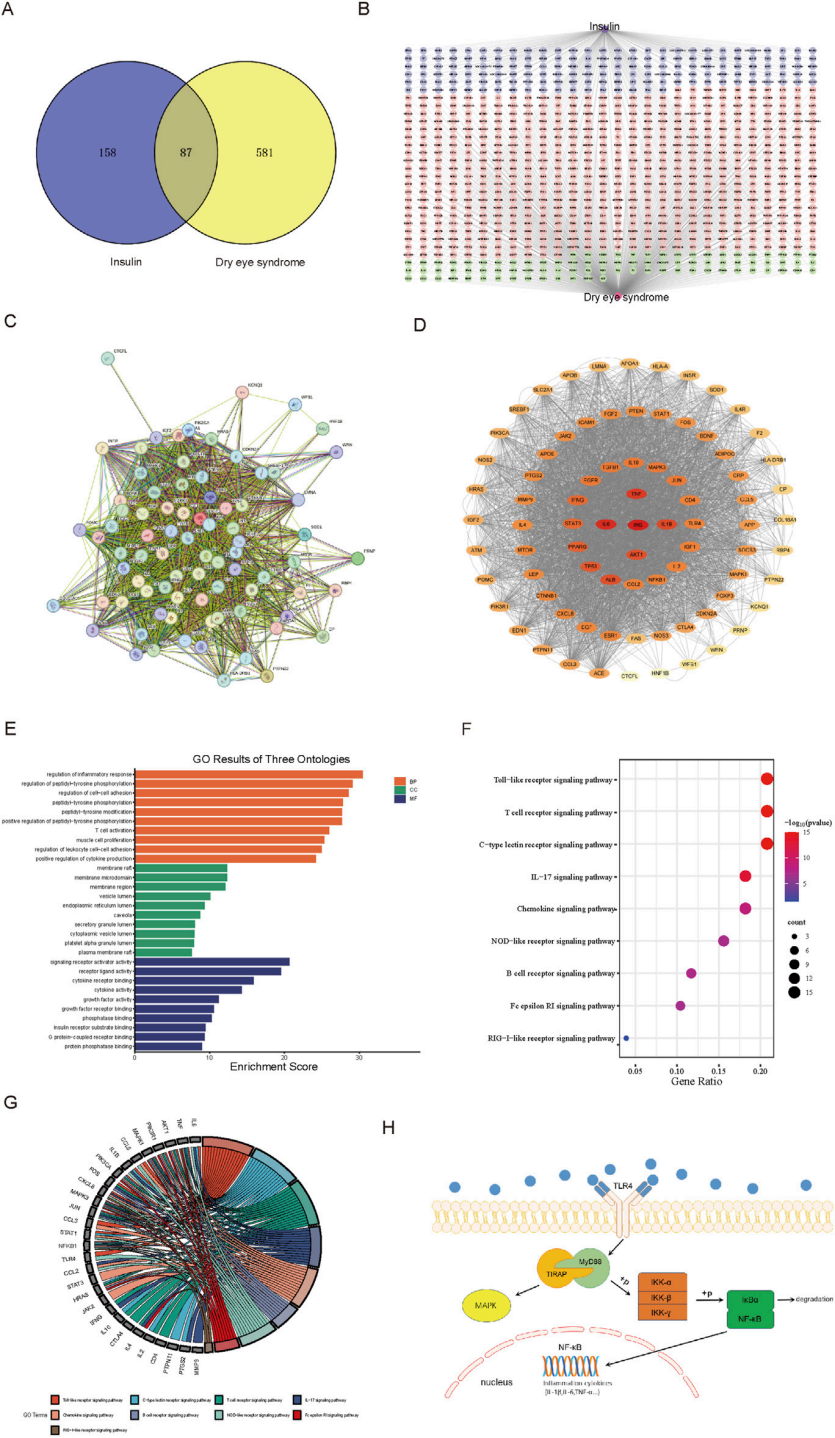
Analysis results based on network pharmacology. **(A)** Venn diagram of insulin and DES targets. **(B)** Network analysis of insulin-target-DES. **(C)** The PPI network was obtained from the STRING 12.0 database platform. **(D)** Visualization of PPI network. **(E)** Bar graph of GO function annotation of overlapping targets. **(F)** Bubble graph for KEGG pathway enrichment analysis of overlapping targets related to the immune system. **(G)** Chordal graph of pathways associated with the immune system and their corresponding targets. **(H)** TLR4 signaling pathway graph.

We submitted 245 insulin and 668 DES targets to the Venny 2.1.0 website, and the Venn diagram (Figure 1A) showed 87 common targets. To screen out the central protein interactions, the respective targets of insulin and DES were imported into Cytoscape 3.9.1 software, and a network graph depicting the interaction between insulin-target-DES was constructed (Figure 1B), which consisted of 828 nodes and 913 edges. The purple area was targets of insulin alone, the pink area was targets of DES alone, and the green part was common targets.

#### 3.1.4 The PPI network of insulin and DES common targets

We entered the 87 common targets of insulin and DES into STRING 12.0, which showed that 81 proteins interacted with each other (Figure 1C). The network of insulin used for DES treatment was constructed by importing these 81 targets into Cytoscape 3.9.1 software (Figure 1D), the network had 81 nodes and 3,686 edges. Then, we calculated the topological indices DC, BC, and CC of these 81 targets. DC suggested the degree of association of a specific target with the targets of insulin action on DES. The higher the DC value, the stronger the association. Darker targets in Figure 1D indicated higher DC values and stronger correlation, and we selected the top ten targets with the highest DC values (Table 1), which may be the core targets for insulin action on DES.

**TABLE 1:** Topology characteristics of hub nodes in the PPI network.

No.	Gene	Protein names	Betweenness Centrality	Degree
1	*INS*	Insulin	0.04651985	150
2	*IL6*	Interleukin-6	0.018757695	146
3	*TNF*	Tumor necrosis factor	0.018757695	142
4	*IL1B*	Interleukin-1 beta	0.013080221	138
5	*AKT1*	Potassium channel AKT1	0.012826321	136
6	*ALB*	Albumin	0.015946646	136
7	*TP53*	Cellular tumor antigen p53	0.021130391	134
8	*PPARG*	Peroxisome proliferator-activated receptor gamma	0.020473824	132
9	*IFNG*	Interferon gamma	0.009662216	130
10	*STAT3*	Signal transducer and activator of transcription 3	0.007079539	130

#### 3.1.5 GO functional annotation and KEGG pathway enrichment analysis

87 insulin and DES common targets were submitted to the Microbiotics Database for GO functional annotation and KEGG pathway enrichment analysis. In the GO functional analysis, BP, CC, and MF were included. There were 2,749 terms associated with BP, 92 terms associated with CC, and 150 terms associated with MF (*P* < 0.05). Each section’s ten most statistically significant results were identified based on their *p*-values and visualized using bar graphs (Figure 1E).

In the KEGG pathway enrichment analysis, 169 pathways were statistically different (*P* < 0.05). Since the immune system plays a pivotal role in DES, we analyzed the immune-related pathways and drew a bubble plot (Figure 1F), which showed that insulin is most likely to be involved in DES through the TLR signaling pathway. Moreover, we produced chordal plots to visualize the relationship between targets and pathways (Figure 1G).

The TLR signaling pathway is an important immunoregulatory pathway, in which TLR4/NF-κB signaling pathway plays a particularly important role in the development of inflammatory diseases such as DES, therefore, our follow-up experiments assessed the effect of insulin on the TLR4/NF-κB signaling pathway, the following figure shows the proteins associated with the TLR4/NF-κB signaling pathway (Figure 1H).

### 3.2 Experimental verification

#### 3.2.1 Cytotoxicity of hyperosmolality and insulin in HCECs

**FIGURE 2:**
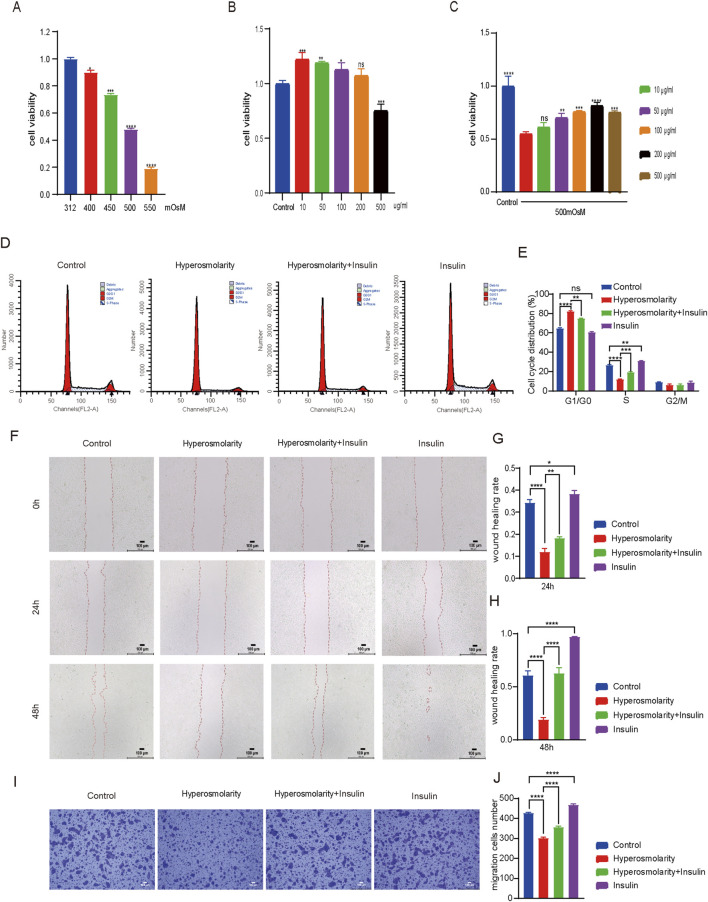
Insulin promoted HCECs proliferation and improved their ability to migrate. **(A–C)** Cell viability of HCECs upon coculture with different osmolalities, **(A)** different concentrations of insulin, **(B)** and osmolarity/insulin combinations **(C)** for 24 h (The control group was treated with complete medium, A vs. 312 mOsM, B vs. control group, C vs. 500 mOsM hyperosmolarity group alone) **(D)**. The cell cycle of HCECs was analyzed by flow cytometry using PI staining. **(E)** Percentage of cell cycle distribution of HCECs. **(F)** Scratch images of 0 h, 24 h and 48 h after HCECs were processed differently. **(G, H)** Quantification analysis of wound healing rate in scratch experiment. **(I)** Transwell assay showed the effect of different treatments on HCECs migration. **(J)** Cell migration number obtained in the transwell assay. n = 3. Data are expressed as the mean ± standard deviation (SD) and analyzed by one-way ANOVA test. (ns = no statistical difference; *P < 0.05; **P < 0.01; ***P < 0.001; ****P < 0.0001).

The viability of HCECs treated with different concentrations of hyperosmolarity or insulin was assessed using the CCK-8 assay to determine the appropriate concentration (Figure 2A–C). For hyperosmolarity tests, cell viability showed a dose-dependent decrease with increasing osmolarity (312–550 mOsM) after 24 h incubation with cells (Figure 2A, *P* < 0.05). The cell viability reached about 50% when the osmotic pressure reached 500 mOsM, so we chose 500 mOsM as the subsequent stimulation concentration. For insulin tests, the results showed that there was no cytotoxicity in the presence of insulin at concentrations from 10 to 200 μg/mL after 24 h incubation with cells (Figure 2B). When the concentration of insulin reached 500 μg/mL, HCECs still showed more than 70% cell viability. (Figure 2B, *P* < 0.01), demonstrating the high biosafety for insulin. Next, we mixed 500mOsM hypertonic solution with insulin (10–500 μg/mL) for 1 day of incubation for further testing (Figure 2C). The results showed that cell viability rose with increasing insulin concentration from 10 to 200 μg/mL, and cell viability was maximized at an insulin concentration of 200 μg/mL (*p* < 0.0001), therefore, in subsequent experiments, the concentration of insulin was selected as 200 μg/mL. The stimulated concentration of hyperosmolarity was selected as 500 mOsM.

#### 3.2.2 The effect of insulin on the cycle and migration of HCECs

Using flow cytometry to investigate further the changes of insulin and hyperosmolarity on the cell cycle (Figures 2D, E). Compared to the control group, the proportion of S-phase cells in the hyperosmolarity group exhibited a significant reduction of 54% (*P* < 0.0001). In comparison, there was a notable increase of 22% (*P* < 0.0001) in the proportion of cells arrested in the G1-G0 phase. In contrast, the hyperosmolarity and insulin co-culture group demonstrated a substantial increase of 38% (*P* < 0.001) in S-phase cells compared with the hyperosmolarity group, accompanied by a decrease of 9% (*P* < 0.01) in cells arrested at G1-G0 phase. No significant changes were observed in the G2/M phase cell population following treatment. The results showed that hyperosmolarity could block the cell cycle of HCECs in the S phase, also known as the DNA synthesis phase. Thus, hyperosmolarity inhibited the proliferation of HCECs by inhibiting DNA synthesis, whereas insulin led to faster DNA synthesis and active proliferation of HCECs.

The effects of insulin and hyperosmolarity on the migratory capacity of HCECs were evaluated using scratch experiments and transwell migration assay (Figures 2F–J). At 0 h, each group of cells formed a scratch of nearly the same area (Figure 2F). After 24 h of treatment, the wound healing rate was 20% lower in the hyperosmolarity group compared to the control group (*P* < 0.0001), while it was approximately 6% higher in the hyperosmolarity and insulin co-culture group compared to the hyperosmolarity group (*P* < 0.01). The trend after 48 h was similar (Figure 2H). Notably, the scratches in the insulin alone treatment group almost disappeared after 48 h (Figure 2F). The results showed hyperosmolarity suppressed the migratory capacity of HCECs, while insulin recovered it.

In the transwell migration experiment (Figures 2I, J), A 30% reduction in cells stained with crystal violet was found in the hyperosmolarity stimulation group compared with the control group (*P* < 0.0001), and a 15% increase in cells stained in the hyperosmolarity and insulin co-culture group compared with the hyperosmolarity group (*P* < 0.0001). The findings further indicated that hyperosmolarity inhibited cellular migration, whereas insulin promoted cell migration.

#### 3.2.3 Insulin inhibited hyperosmolarity-induced ROS production and apoptosis

**FIGURE 3:**
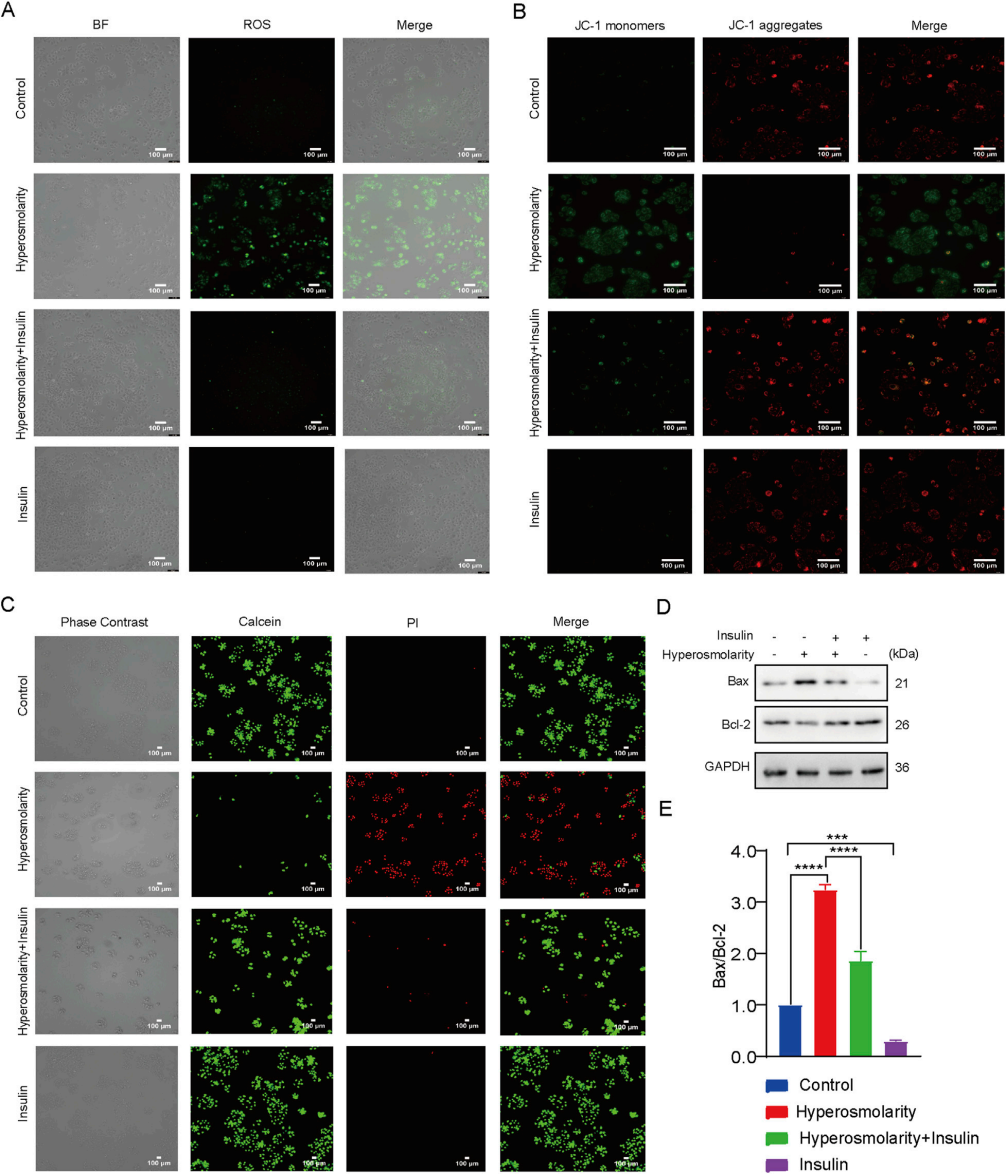
Insulin inhibited hyperosmolarity-induced apoptosis. **(A)** Intracellular ROS levels determined using DCFH-DA staining. **(B)** Determination of mitochondrial membrane potential by JC-1. **(C)** Calcein/PI staining images of HCECs with different treatments. **(D)** Expression of Bax and Bcl-2 proteins detected by western blot. **(E)** The ratio of Bax/Bcl-2 protein expression. n = 3. Data are expressed as the mean ± standard deviation (SD) and analyzed by one-way ANOVA test. (ns = no statistical difference; *P < 0.05; **P < 0.01; ***P < 0.001; ****P < 0.0001).

ROS generation was investigated using DCFH-DA staining. Intracellular ROS have the ability to oxidize non-fluorescent DCFH to fluorescent DCF. Monitoring the fluorescence intensity of DCF enables the quantification of intracellular ROS levels. Green fluorescence was barely detectable in the control and insulin-alone groups, with the majority of cells in the hyperosmolarity group showing green fluorescence, which was attenuated in the hyperosmolarity and insulin co-culture group (Figure 3A). The results demonstrated that hyperosmolarity treatment produced a large amount of ROS, whereas insulin reduced ROS production.

We examined alterations in mitochondrial membrane potential (MMP) using the JC-1 assay kit. Red fluorescence is generated in response to a high MMP, whereas green fluorescence is produced when the MMP is low. This makes it very convenient to detect changes in MMP by changing the fluorescence color. It can be seen that the cells in the control and insulin alone groups showed predominantly red fluorescence, most of the cells in the hyperosmolarity-treated group showed green fluorescence, and the proportion of cells producing green fluorescence in the hyperosmolarity and insulin co-culture group was significantly diminished (Figure 3B). This suggested that hyperosmolarity stimulation decreased MMP, and enhanced mitochondrial damage. In addition, insulin was also demonstrated to be a potential antioxidant that may inhibit oxidative stress by improving mitochondrial function.

The decrease in cell membrane potential can be an early indicator of apoptosis detection. Next, we detected apoptosis with the Calcein/PI assay (Figure 3C). The Calcein AM labels live cells with green fluorescence. In contrast, dead cells are stained with red fluorescence using Propidium Iodide (PI). Therefore, combined with PI, Calcein AM can be used for simultaneous double fluorescence staining of both live and dead cells to detect cell activity and cytotoxicity. Cells in both the control and insulin alone groups emitted green solid fluorescence. In contrast, cells in the hyperosmolarity group emitted strong red fluorescence, and the percentage of cells producing red fluorescence was significantly reduced in the hyperosmolarity and insulin co-culture group (Figure 3C). The results demonstrated that hyperosmolarity promoted apoptosis, whereas insulin inhibited it.

Western blot experiments (Figures 3D,E) revealed that the ratio of Bax/Bcl-2 protein expression in the hyperosmolarity stimulation group was more than three times that of the control group (*P* < 0.0001). In comparison to the hyperosmolarity group, the ratio of Bax/Bcl-2 protein expression decreased by 43% (*P* < 0.0001) in the hyperosmolarity and insulin co-culture group. The above results suggested that hyperosmolarity may promote apoptosis in HCECs via the mitochondrial pathway, while insulin prevented this alteration.

#### 3.2.4 Insulin reduced the expression of inflammatory cytokines in HCECs exposed to hyperosmolarity

**FIGURE 4:**
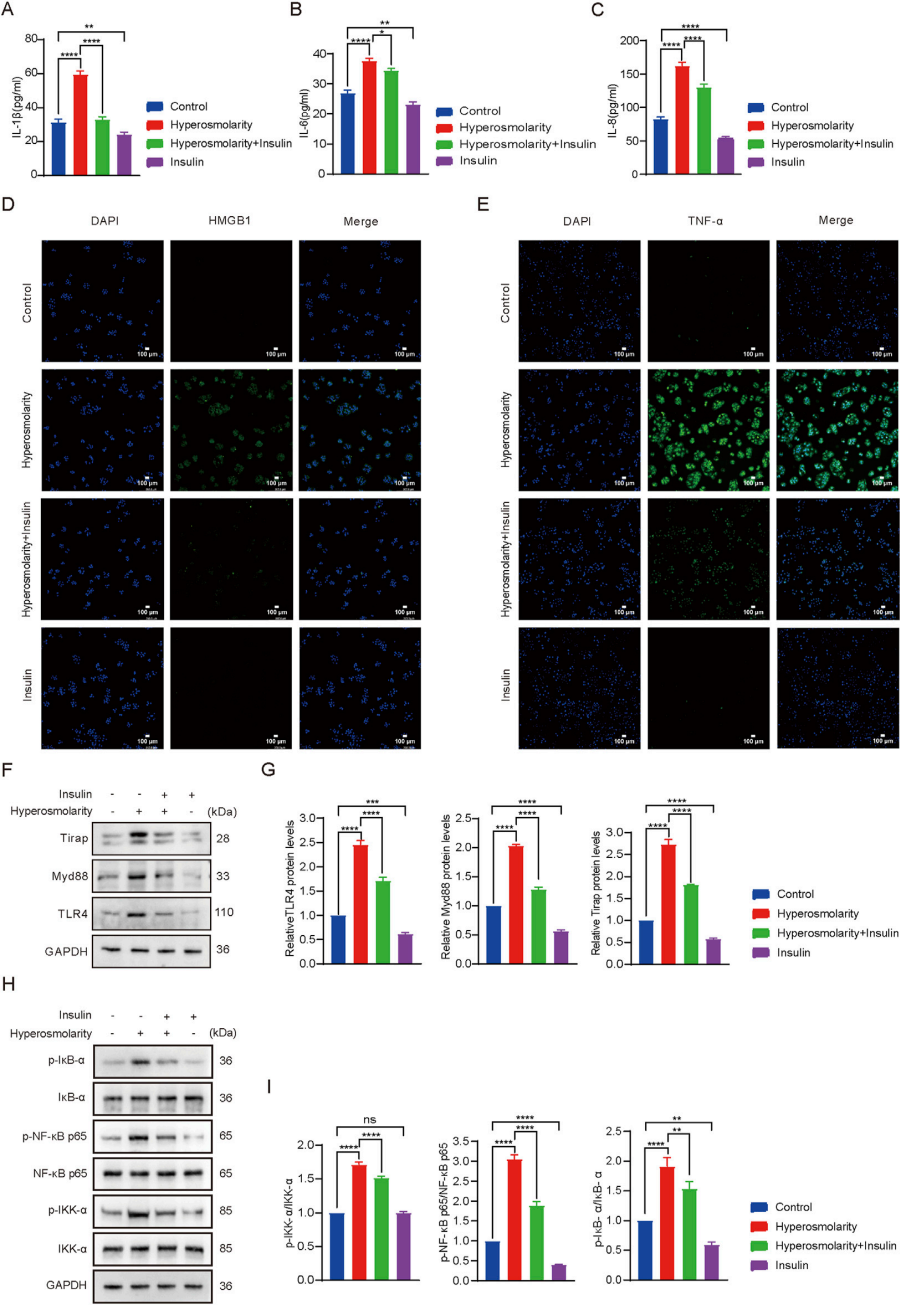
Insulin inhibited the production of hyperosmolarity-induced inflammatory factors and activation of theTLR4 signaling pathway. IL-1β **(A)**, IL-6 **(B)** and IL-8 **(C)** production determined using ELISA assay. Detection of HMGB1 **(D)** and TNF-α **(E)** production using immunofluorescence staining assay. **(F)** Expression of TLR4, MyD88 and Tirap proteins. **(G)** Relative protein levels of TLR4, MyD88 and Tirap. **(H)** Expression of IKK-α, p-IKK-α, NF-κB p65, p-NF-κB p65, IκB-α, p-IκB-α proteins. **(I)** R]elative protein levels of IKK-α, p-IKK-α, NF-κB p65, p-NF-κB p65, IκB-α, p-IκB-α. n = 3. Data are expressed as the mean ± standard deviation (SD) and analyzed by one-way ANOVA test. (ns = no statistical difference; *P < 0.05; **P < 0.01; ***P < 0.001; ****P < 0.0001).

IL-1β, IL-6, and IL-8 levels were measured using the ELISA (Figures 4A–C). Compared to the control group, hyperosmolarity stimulation increased levels of IL-1β, IL-6, and IL-8 by 50% (*P* < 0.0001), 30% (*P* < 0.0001), and 50% (*P* < 0.0001), respectively. Compared with the hyperosmolarity group, the hyperosmolarity and insulin co-culture group reduced their expression levels by 46% (*P* < 0.0001), 11% (*P* < 0.05) and 20% (*P* < 0.0001), respectively. The results demonstrated that hyperosmolarity increased the expression levels of inflammatory cytokines, however, insulin downregulated the levels of inflammatory factors and had an anti-inflammatory effect.

High-mobility group box 1 (HMGB1) is a member of the “alarmins” family that can localize intracellular inflammatory mediators in the resting state (Abdelmageed and Abdelrahman, 2023). When released, HMGB1 triggers a pro-inflammatory cascade and is involved in cellular damage (Qi et al., 2021). Next, immunofluorescence was performed to test the expression of HMGB1 and TNF-α proteins in HCECs (Figures 4D,E). Fluorescence detection of HMGB1 protein showed that green fluorescence was barely detectable in the control and insulin alone groups. Most of the cells in the hyperosmolarity-stimulated group produced green fluorescence, which was attenuated in the hyperosmolarity and insulin co-culture group (Figure 4D). The same was valid for changes in fluorescence intensity of TNF-α proteins (Figure 4E). The results demonstrated that hyperosmolarity promoted the release of the pro-inflammatory factor HMGB1 and induced the expression of the inflammatory factor TNF-α, whereas insulin inhibited the expression of HMGB1 and TNF-α proteins. It further illustrated the essential biological function of insulin as an anti-inflammatory.

#### 3.2.5 Insulin inhibited the activation of the TLR4/NF-κB signaling pathway

The impact of insulin on the TLR4/NF-κB signaling pathway was evaluated using western blot analysis (Figures 4F–I). TLR4, MyD88, and Tirap expression levels in the hyperosmolarity group were 2.4 times, 2 times, and 2.7 times that of the control group, respectively (*P* < 0.0001). Protein expression levels were reduced by 33%, 40%, and 32% in the hyperosmolarity and insulin co-culture group, respectively, compared with the hyperosmolarity group (*P* < 0.0001). After hyperosmolarity treatment, the degree of phosphorylation of IKK-α, NF-κB p65 and IκB-α increased significantly (Figures 4H, I), and the expression of p-IKK-α, p-NF-κB p65 and p-IκB-α in the hyperosmolarity group was 1.7 times, 1.9 times and 3 times that of the control group, respectively (*P* < 0.0001). Compared with the hyperosmolarity group, protein expression decreased by 12%, 21%, and 40% in the hyperosmolarity and insulin co-culture group (*P* < 0.01), respectively. The results demonstrated that insulin effectively suppressed the expression of proteins involved TLR4/NF-κB signaling pathway.

## 4 Discussion

The management of DES represents a significant global healthcare challenge in the field of ophthalmology (Hong et al., 2020). It has long been recognized as a multifactorial, complex condition caused by decreased tear production or excessive evaporation, ultimately leading to hyperosmolar tears (Huang et al., 2022). The primary pathogenesis of this condition involves chronic inflammation of the ocular surface. The induction of inflammatory responses in the *in vitro* culture can be achieved through the use of a hypertonic medium (500 mOsM). In addition, benzalkonium chloride (BAC) (1 μg/mL), IL-1β (10 ng/mL), and TNF-α (10 ng/mL) can be used to induce a cascade reaction of inflammation (Rahman et al., 2021). However, for DES, hypertonic tears are believed to serve as the inciting stimulus for initiating inflammation, which induce inflammatory responses on the ocular surface by activating HCECs. Therefore, 500 mOsM of hypertonic medium was used in this study to induce the inflammatory response in DES. A previous study chose 470 mOsM as the most appropriate stimulation concentration (López-Cano et al., 2021), and the reason for this discrepancy may be the differences in osmolality measuring instruments, the influence of the experimental environment and personal operation, and so on.

In addition to regulating blood glucose levels, insulin has the ability to regulate the activity of biological enzymes, which in turn promotes cell growth and regulates the transcription of specific genes, and plays an important role in maintaining and controlling cell growth, proliferation, differentiation, maturation and regeneration (Duan et al., 2021; Khodabakhsh et al., 2021; Burgos-Blasco et al., 2023). Its use in wound healing in nondiabetic patients has attracted increasing attention, and clinical studies have quizzed its use in the cornea, finding that the efficacy of insulin in promoting wound healing is not dependent on the regulation of blood glucose levels (Diaz-Valle et al., 2022). In addition, insulin can suppress the production of inflammatory cytokines and oxidative stress, which has a therapeutic effect on chronic inflammatory diseases (Dallak et al., 2019). In this study, we explored the potential mechanism of insulin action on DES by network pharmacology and verified the specific effect of insulin by cellular experiments.

We screened 87 targets of insulin action on DES by a network pharmacology approach, suggesting that insulin exerts protective effects on DES through multiple targets. Moreover, we constructed a PPI network of insulin and DES common targets and performed GO functional annotation and KEGG pathway enrichment analysis of the common targets. The results suggested that insulin was most likely involved in the development of DES through the TLR signaling pathway.

Insulin is water-soluble, and we put it in weakly acidic PBS. Our preliminary tests confirmed that insulin at concentrations less than 500 μg/mL was nontoxic (cell viability >75%) for HCECs. The highest cell viability was achieved at an insulin concentration of 200 μg/mL when mixed with 500mOsM hypertonic medium for 24 h. To investigate the impact of insulin on cellular biological functionality, we co-cultured HCECs with insulin or hyperosmolarity medium by flow assay, cell scratch assay, and transwell migration assay, respectively. The analysis revealed that insulin significantly enhanced the migratory capacity of HCECs inhibited by hyperosmolarity and promoted HCECs proliferation.

Pathophysiologic processes involved in DES include short tear film breakup time, tear hyperosmolarity, oxidative stress, and apoptosis (I Y Hasan, 2021). ROS are potent oxidizing agents found in aerobic organisms, including oxygen radicals and their derivatives (Herb and Schramm, 2021). The production of ROS results in a reduction in MMP, which is also considered a pivotal event during the initial phases of apoptosis (You et al., 2018). Therefore, we examined the effects of insulin on ROS production, MMP, and apoptosis of HCECs. The results showed that insulin reduced ROS production, increased MMP, and inhibited apoptosis. Moreover, western blot experiments revealed that insulin decreased the expression of the pro-apoptotic protein Bax and upregulated the expression of the anti-apoptotic protein Bcl-2. These results suggested that insulin may inhibit apoptosis in HCECs by suppressing oxidative stress and improving mitochondrial function. Consistently, Allen et al. suggested that insulin treatment can inhibit cell apoptosis in vascular smooth muscle cells (Allen et al., 2005). Viardot et al. determined that insulin protected the mitochondrial ultrastructure of hepatocytes, maintained normal mitochondrial function, and inhibited cell apoptosis (Viardot et al., 2007). In addition, insulin inhibited oxidative stress by improving mitochondrial function or inhibiting cytokine release in septic patients and animals (Kun et al., 2015; Chen et al., 2018).

Although the pathogenesis of DES remains incompletely understood, inflammation, as a result of early innate immune and adaptive responses, has been identified as a crucial contributor that can initiate the vicious cycle of DES (Hakim and Farooq, 2022). In ELISA and immunofluorescence experiments, we found that hypertonic solution (500 mOsM) induced an increase in the protein expression of the proinflammatory factor HMGB1, and also caused significant overexpression of the inflammatory factors IL- 1β, IL-6, IL-8, and TNF-α, which led to an increase in the inflammatory damage in HCECs. The expression of the above factors decreased significantly after insulin intervention, which in turn prevented further exacerbation of the inflammatory response. Similarly, Jeschke et al. (2002). found that treatment with insulin after thermal injury decreased pro-inflammatory cytokines and their intracellular signaling, increased anti-inflammatory cytokines and their intracellular signaling (Jeschke et al., 2002). Previous studies have demonstrated the inhibitory effect of insulin on the release of sepsis-associated cytokines, including IL-1, IL-6, and TNF-α (Zou et al., 2012). Moreover, it was confirmed to suppress the transcription factor NF-κB in human aortic endothelial cells *in vitro*, thereby downregulating the expression of pro-inflammatory cytokines (Aljada and Dandona, 2000). All of these findings confirmed that insulin could inhibit the expression of inflammatory and pro-inflammatory factors and was a good anti-inflammatory agent.

TLRs are membrane-binding proteins and are crucial for initiating and regulating the immune response (Akira, 2003). Among the family of TLRs, TLR4 is the first toll-like receptor to be discovered and is one of the TLRs closely associated with immune inflammation. Experimental studies have shown that TLR4 was significantly expressed in the inflammatory process of DES. After TLR4 expression was inhibited, the inflammatory response and tissue damage were reduced (Hoshino et al., 2016; Ju et al., 2018). TLR4 can induce NF-κB activation and cytokine production via MyD88 protein. Upon activation of TLR4, IκB molecules are phosphorylated through the MyD88-dependent pathway, which activates the downstream NF-κB signaling pathway and ultimately induces the expression of inflammatory factors in host cells.

Some studies found that TLR4/NF-κB pathway proteins were highly expressed in autoimmune uveitis (Liu et al., 2022; He et al., 2023),another study showed that the TLR4/NF-κB pathway was activated in high IOP-induced retinal ganglion cell damage (Shangli et al., 2024), TLR4/NF-κB pathway-associated proteins were also highly expressed in retinal inflammation caused by microglia activation (Han et al., 2020). Moreover, the upregulation of TLR4 and downstream NF-κB signaling proteins was observed in DES (Lee et al., 2012; Redfern et al., 2015; Simmons et al., 2016; Zhang et al., 2019; Yang et al., 2021). With similar results to the above, in western blot experiments, we also observed upregulation of TLR4/NF-κB signaling pathway proteins in HCECs after hyperosmotic treatment and the expression of TLR4/NF-κB signaling pathway proteins could be significantly reduced after insulin action. This suggested that insulin may play a protective role against DES by inhibiting the activation of the TLR4/NF-κB signaling pathway.

Our study has several limitations, because genetic databases may be incomplete, which may reduce the confidence of predictions. This paper also only validates the *in vitro* model of DES. At the same time, the effect of hypertonic medium on cell death was not ruled out in cell characterization experiments (e.g., scratch experiments, migration experiments, etc.). Nevertheless, our findings suggest that insulin may be a promising candidate for treating DES.

## 5 Conclusion

Our results suggested that insulin could promote cell proliferation, improve cell migration ability, reduce ROS production, increase mitochondrial membrane potential, and inhibit apoptosis. Moreover, insulin also inhibited the production of inflammatory factors and the activation the TLR4/NF-κB signaling pathway. It provides a valuable reference for the clinical management of DES.

## Data Availability

The datasets presented in this study can be found in online repositories. The names of the repository/repositories and accession number(s) can be found in the article/supplementary material. Further inquiries can be directed to the corresponding author.

## References

[B1] Abdelmageed M. E. Abdelrahman R. S. (2023). Canagliflozin attenuates thioacetamide-induced liver injury through modulation of HMGB1/RAGE/TLR4 signaling pathways. Life Sci. 322, 121654. 10.1016/j.lfs.2023.121654 37023955

[B2] Akira S. (2003). Toll-like receptor signaling. J. Biol. Chem. 278 (40), 38105–38108. 10.1074/jbc.R300028200 12893815

[B3] Aljada A. Dandona P. (2000). Effect of insulin on human aortic endothelial nitric oxide synthase. Metabolism-Clinical Exp. 49 (2), 147–150. 10.1016/s0026-0495(00)91039-4 10690935

[B4] Allen R. T. Krueger K. D. Dhume A. Agrawal D. K. (2005). Sustained Akt/PKB activation and transient attenuation of c-jun N-terminal kinase in the inhibition of apoptosis by IGF-1 in vascular smooth muscle cells. Apoptosis 10 (3), 525–535. 10.1007/s10495-005-1882-3 15909115

[B5] An J. S. Fan H. L. Han M. Y. Peng C. Xie J. Peng F. (2022). Exploring the mechanisms of neurotoxicity caused by fuzi using network pharmacology and molecular docking. Front. Pharmacol. 13, 961012. 10.3389/fphar.2022.961012 36110545 PMC9468872

[B6] Burgos-Blasco B. Diaz-Valle D. Rego-Lorca D. Perez-Garcia P. Puebla-Garcia V. Fernandez-Vigo J. I. (2023). Topical insulin, a novel corneal epithelial regeneration agent in dry eye disease. Eur. J. Ophthalmol. 7, 719–725. 10.1177/11206721231206790 37814519

[B7] Chang Y. W. Hung L. C. Chen Y. C. Wang W. H. Lin C. Y. Tzeng H. H. (2021). Insulin reduces inflammation by regulating the activation of the NLRP3 inflammasome. Front. Immunol. 11, 587229. 10.3389/fimmu.2020.587229 33679687 PMC7933514

[B8] Chen G. D. Zhang J. L. Chen Y. T. Zhang J. X. Wang T. Zeng Q. Y. (2018). Insulin alleviates mitochondrial oxidative stress involving upregulation of superoxide dismutase 2 and uncoupling protein 2 in septic acute kidney injury. Exp. Ther. Med. 15 (4), 3967–3975. 10.3892/etm.2018.5890 29563990 PMC5858081

[B9] Chen Q. Y. Yu W. K. Shi J. L. Shen J. H. Gao T. Zhang J. J. (2014). Insulin alleviates the inflammatory response and oxidative stress injury in cerebral tissues in septic rats. J. Inflammation-London 11, 18. 10.1186/1476-9255-11-18 PMC4108965 25093012

[B10] Chen Y. Z. Chen Z. Y. Tang Y. J. Tsai C. H. Chuang Y. L. Hsieh E. H. (2021). Development of lutein-containing eye drops for the treatment of dry eye syndrome. Pharmaceutics 13 (11), 1801. 10.3390/pharmaceutics13111801 34834216 PMC8621052

[B11] Conart J. B. Blot G. Augustin S. Millet-Puel G. Roubeix C. Beguier F. (2020). Insulin inhibits inflammation-induced cone death in retinal detachment. J. Neuroinflammation 17 (1), 358. 10.1186/s12974-020-02039-1 33243251 PMC7694924

[B12] Cruz-Cazarim E. L. C. Cazarim M. S. Ogunjimi A. T. Petrilli R. Rocha E. M. Lopez R. F. V. (2019). Prospective insulin-based ophthalmic delivery systems for the treatment of dry eye syndrome and corneal injuries. Eur. J. Pharm. Biopharm. 140, 1–10. 10.1016/j.ejpb.2019.04.014 31015020

[B13] Dallak M. Al-Ani B. Kader D. H. A. Eid R. A. Haidara M. A. (2019). Insulin suppresses type 1 diabetes mellitus-induced ventricular cardiomyocyte damage associated with the inhibition of biomarkers of inflammation and oxidative stress in rats. Pharmacology 104 (3-4), 157–165. 10.1159/000500898 31185481

[B14] Diaz-Valle D. Burgos-Blasco B. Rego-Lorca D. Puebla-Garcia V. Perez-Garcia P. Benitez-del-Castillo J. M. (2022). Comparison of the efficacy of topical insulin with autologous serum eye drops in persistent epithelial defects of the cornea. Acta Ophthalmol. 100 (4), E912–E919. 10.1111/aos.14997 34407296

[B15] Duan H. M. Feng X. T. Huang X. Q. (2021). Effects of insulin on the proliferation and global gene expression profile of A7r5 cells. Mol. Biol. Rep. 48 (2), 1205–1215. 10.1007/s11033-021-06200-8 33555531

[B16] Ferreira S. S. Oliveira M. A. Tsujita M. Nunes F. P. B. Casagrande F. B. Gomes E. (2020). Insulin modulates the immune cell phenotype in pulmonary allergic inflammation and increases pulmonary resistance in diabetic mice. Front. Immunol. 11, 84. 10.3389/fimmu.2020.00084 32117245 PMC7026190

[B17] Hakim F. E. Farooq A. V. (2022). Dry eye disease an update in 2022. Jama-Journal Am. Med. Assoc. 327 (5), 478–479. 10.1001/jama.2021.19963 35103781

[B18] Han X. K. Chen X. Chen S. L. Luo Q. Liu X. He A. Q. (2020). Tetramethylpyrazine attenuates endotoxin-induced retinal inflammation by inhibiting microglial activation via the TLR4/NF-κB signalling pathway. Biomed. and Pharmacother. 128, 110273. 10.1016/j.biopha.2020.110273 32460188

[B19] He S. Y. Li W. Q. Wang G. Q. Wang X. T. Fan W. Zhang Z. (2023). FTO-mediated m6A modification alleviates autoimmune uveitis by regulating microglia phenotypes via the GPC4/TLR4/NF-κB signaling axis. Genes and Dis. 10 (5), 2179–2193. 10.1016/j.gendis.2022.09.008 PMC10363593 37492748

[B20] Herb M. Schramm M. (2021). Functions of ROS in macrophages and antimicrobial immunity. Antioxidants 10 (2), 313. 10.3390/antiox10020313 33669824 PMC7923022

[B21] Hong S. C. Ha J. H. Lee J. K. Jung S. H. Kim J. C. (2020). *In vivo* anti-inflammation potential of *Aster koraiensis* extract for dry eye syndrome by the protection of ocular surface. Nutrients 12 (11), 3245. 10.3390/nu12113245 33113960 PMC7690718

[B22] Hoshino K. Takeuchi O. Kawai T. Sanjo H. Ogawa T. Takeda Y. (2016). Cutting edge: toll-like receptor 4 (TLR4)-Deficient mice are hyporesponsive to lipopolysaccharide: evidence for TLR4 as the *lps* gene product. J. Immunol. 197 (7), 3749–3752. 10.4049/jimmunol.162.7.3749 10201887

[B23] Huang R. J. Su C. Y. Fang L. Lu J. Q. Chen J. S. Ding Y. (2022). Dry eye syndrome: comprehensive etiologies and recent clinical trials. Int. Ophthalmol. 42 (10), 3253–3272. 10.1007/s10792-022-02320-7 35678897 PMC9178318

[B24] I Y Hasan Z. A. (2021). Dry eye syndrome risk factors: A systemic review. Saudi J. Ophthalmol. official J. Saudi Ophthalmol. Soc. 35 (2), 131–139. 10.4103/1319-4534.337849 PMC8982940 35391807

[B25] Jeschke M. G. Einspanier R. Klein D. Jauch K. W. (2002). Insulin attenuates the systemic inflammatory response to thermal trauma. Mol. Med. 8 (8), 443–450. 10.1007/bf03402024 12435855 PMC2040014

[B26] Jin R. Li Y. Li L. Kim D. H. Yang C. D. Son H. S. (2020). Anti-inflammatory effects of glycine thymosin β4 eye drops in experimental dry eye. Biomed. Rep. 12 (6), 319–325. 10.3892/br.2020.1296 32382416 PMC7201140

[B27] Ju M. J. Liu B. F. He H. Y. Gu Z. Y. Liu Y. M. Su Y. (2018). MicroRNA-27a alleviates LPS-induced acute lung injury in mice via inhibiting inFLammation and apoptosis through modulating TLR4/MyD88/NF-κB pathway. Cell Cycle 17 (16), 2001–2018. 10.1080/15384101.2018.1509635 30231673 PMC6260216

[B28] Khodabakhsh P. Pournajaf S. Shalmani L. M. Ahmadiani A. Dargahi L. (2021). Insulin promotes schwann-like cell differentiation of rat epidermal neural crest stem cells. Mol. Neurobiol. 58 (10), 5327–5337. 10.1007/s12035-021-02423-9 34297315

[B29] Kiripolsky J. Romano R. A. Kasperek E. M. Yu G. Kramer J. M. (2020). Activation of myd88-dependent TLRs mediates local and systemic inflammation in a mouse model of primary sjogren's syndrome. Front. Immunol. 10, 2963. 10.3389/fimmu.2019.02963 31993047 PMC6964703

[B30] Kong X. B. Liu C. A. X. Lu P. Guo Y. Z. Zhao C. C. Yang Y. Y. (2021). Combination of UPLC-Q-TOF/MS and network pharmacology to reveal the mechanism of qizhen decoction in the treatment of colon cancer. Acs Omega 6 (22), 14341–14360. 10.1021/acsomega.1c01183 34124457 PMC8190929

[B31] Kun S. Molnár G. A. Sélley E. Szélig L. Bogár L. Csontos C. (2015). Insulin therapy of nondiabetic septic patients is predicted by *para*-tyrosine/phenylalanine ratio and by hydroxyl radical-derived products of phenylalanine. Oxidative Med. Cell. Longev. 7, 839748. 10.1155/2015/839748 PMC4630663 26576228

[B32] Lee H. S. Hattori T. Park E. Y. Stevenson W. Chauhan S. K. Dana R. (2012). Expression of toll-like receptor 4 contributes to corneal inflammation in experimental dry eye disease. Investigative Ophthalmol. and Vis. Sci. 53 (9), 5632–5640. 10.1167/iovs.12-9547 PMC3711695 22789921

[B33] Liao Y. K. Jiang H. T. Du Y. R. Xiong X. J. Zhang Y. Du Z. Y. (2022). Using convolutional neural network as a statistical algorithm to explore the therapeutic effect of insulin liposomes on corneal inflammation. Comput. Intell. Neurosci. 2022, 1169438. 10.1155/2022/1169438 35958780 PMC9357760

[B34] Liu Y. S. Zhao C. Y. Meng J. Y. Li N. Xu Z. R. Liu X. Y. (2022). Galectin-3 regulates microglial activation and promotes inflammation through TLR4/MyD88/NF-kB in experimental autoimmune uveitis. Clin. Immunol. 236, 108939. 10.1016/j.clim.2022.108939 35121106

[B35] López-Cano J. J. González-Cela-Casamayor M. A. Andres-Guerrero V. Herrero-Vanrell R. Benítez-Del-Castillo J. M. Molina-Martínez I. T. (2021). Combined hyperosmolarity and inflammatory conditions in stressed human corneal epithelial cells and macrophages to evaluate osmoprotective agents as potential DED treatments. Exp. Eye Res. 211, 108723. 10.1016/j.exer.2021.108723 34384756

[B36] Moshirfar M. Pierson K. Hanamaikai K. Santiago-Caban L. Muthappan V. Passi S. F. (2014). Artificial tears potpourri: a literature review. Clin. Ophthalmol. 8, 1419–1433. 10.2147/opth.S65263 25114502 PMC4124072

[B37] Pan S. S. Wang F. Hui Y. P. Chen K. Y. Zhou L. Gao W. L. (2023). Insulin reduces pyroptosis-induced inflammation by PDHA1 dephosphorylation-mediated NLRP3 activation during myocardial ischemia-reperfusion injury. Perfusion-Uk 38 (6), 1277–1287. 10.1177/02676591221099807 35506656

[B38] Qi X. W. Wang H. G. Xia L. C. Lin R. W. Li T. Guan C. N. (2021). miR-30b-5p releases HMGB1 via UBE2D2/KAT2B/HMGB1 pathway to promote pro-inflammatory polarization and recruitment of macrophages. Atherosclerosis 324, 38–45. 10.1016/j.atherosclerosis.2021.02.016 33812169

[B39] Rahman M. M. Kim D. H. Park C. K. Kim Y. H. (2021). Experimental models, induction protocols, and measured parameters in dry eye disease: focusing on practical implications for experimental research. Int. J. Mol. Sci. 22 (22), 12102. 10.3390/ijms222212102 34830010 PMC8622350

[B40] Redfern R. L. Barabino S. Baxter J. Lema C. McDermott A. M. (2015). Dry eye modulates the expression of toll-like receptors on the ocular surface. Exp. Eye. Res. 134, 80–89. 10.1016/j.exer.2015.03.018 25817729 PMC5587194

[B41] Sahoo B. R. (2020). Structure of fish Toll-like receptors (TLR) and NOD-like receptors (NLR). Int. J. Biol. Macromol. 161, 1602–1617. 10.1016/j.ijbiomac.2020.07.293 32755705 PMC7396143

[B42] Shang L. R. Wang Y. C. Li J. X. Zhou F. Y. Xiao K. M. Liu Y. H. (2023). Mechanism of Sijunzi Decoction in the treatment of colorectal cancer based on network pharmacology and experimental validation. J. Ethnopharmacol. 302, 115876. 10.1016/j.jep.2022.115876 36343798

[B43] Shangli J. Yanfang P. Jian L. Pang X. Shibo T. (2024). Human adipose tissue-derived stem cell extracellular vesicles attenuate ocular hypertension-induced retinal ganglion cell damage by inhibiting microglia- TLR4/MAPK/NF-κB proinflammatory cascade signaling. Acta Neuropathol. Commun. 12 (1), 44. 10.1186/s40478-024-01753-8 38504301 PMC10953184

[B44] Sheppard J. Shen Lee B. Periman L. M. (2023). Dry eye disease: identification and therapeutic strategies for primary care clinicians and clinical specialists. Ann. Med. 55 (1), 241–252. 10.1080/07853890.2022.2157477 36576348 PMC9809411

[B45] Simmons K. T. Xiao Y. Y. Pflugfelder S. C. de Paiva C. S. (2016). Inflammatory response to lipopolysaccharide on the ocular surface in a murine dry eye model. Investigative Ophthalmol. Vis. Sci. 57 (6), 2443–2451. 10.1167/iovs.15-18396 PMC4857831 27136463

[B46] Vanhorebeek I. De Vos R. Mesotten D. Wouters P. J. De Wolf-Peeters C. Van den Berghe G. (2005). Protection of hepatocyte mitochondrial ultrastructure and function by strict blood glucose control with insulin in critically ill patients. Lancet 365 (9453), 53–59. 10.1016/s0140-6736(04)17665-4 15639679

[B47] Viardot A. Grey S. T. Mackay F. Chisholm D. (2007). Potential antiinflammatory role of insulin via the preferential polarization of effector T cells toward a T helper 2 phenotype. Endocrinology 148 (1), 346–353. 10.1210/en.2006-0686 17008395

[B48] Yang F. M. Fan D. Yang X. Q. Zhu F. H. Shao M. J. Li Q. (2021). The artemisinin analog SM934 alleviates dry eye disease in rodent models by regulating TLR4/NF-κB/NLRP3 signaling. Acta Pharmacol. Sin. 42 (4), 593–603. 10.1038/s41401-020-0484-5 32747720 PMC8114933

[B49] Yang S. Zhang Y. Y. Zhang Z. H. Dan J. Zhou Q. J. Wang X. C. (2020). Insulin promotes corneal nerve repair and wound healing in type 1 diabetic mice by enhancing wnt/β-catenin signaling. Am. J. Pathology 190 (11), 2237–2250. 10.1016/j.ajpath.2020.08.006 32858016

[B50] You P. T. Wu H. Z. Deng M. Peng J. L. Li F. P. Yang Y. F. (2018). Brevilin A induces apoptosis and autophagy of colon adenocarcinoma cell CT26 via mitochondrial pathway and PI3K/AKT/mTOR inactivation. Biomed. and Pharmacother. 98, 619–625. 10.1016/j.biopha.2017.12.057 29289836

[B51] Zhang X. Z. Yin Y. Yue L. Gong L. (2019). Selective serotonin reuptake inhibitors aggravate depression-associated dry eye via activating the NF-κB pathway. Investigative Ophthalmol. and Vis. Sci. 60 (1), 407–419. 10.1167/iovs.18-25572 30695093

[B52] Zou B. Chen Q. Y. Tang S. Q. Gao T. Zhang J. J. Xi F. C. (2012). Timing of insulin therapy affects the inflammatory response in endotoxemic rats. Inflammation 35 (2), 723–729. 10.1007/s10753-011-9367-8 21809046

